# Diagnostic ability of inner macular layers to discriminate early glaucomatous eyes using vertical and horizontal B-scan posterior pole protocols

**DOI:** 10.1371/journal.pone.0198397

**Published:** 2018-06-07

**Authors:** Maria P. Bambo, Beatriz Cameo, Ruben Hernandez, Enrique Fuentemilla, Noemi Güerri, Blanca Ferrandez, Vicente Polo, Jose M. Larrosa, Luis E. Pablo, Elena Garcia-Martin

**Affiliations:** 1 Ophthalmology Department, Miguel Servet University Hospital, Zaragoza, Spain; 2 Miguel Servet Ophthalmology Innovative and Research Group (GIMSO), Aragon Institute for Health Research (IIS Aragon), Zaragoza, Spain; 3 University of Zaragoza, Zaragoza, Spain; Bascom Palmer Eye Institute, UNITED STATES

## Abstract

**Purpose:**

To evaluate the diagnostic ability of macular ganglion cell (mGCL) and macular retinal nerve fiber (mRNFL) layers, to detect early glaucomatous eyes, using the new segmentation software of Spectralis optical coherence tomography (OCT) device (Heidelberg Engineering).

**Methods:**

A total of 83 eyes from 83 subjects were included in this observational, prospective cross-sectional study: 43 healthy controls and 40 early primary open-angle glaucoma (POAG) patients. All participants were examined using the Horizontal and Vertical Posterior Pole protocols, and the peripapillary RNFL (pRNFL) protocol of Spectralis OCT device. The new automated retinal segmentation software was applied to horizontal and vertical macular B-scans to determine mGCL and mRNFL thicknesses in each one of the 9 sectors of the Early Treatment Diagnostic Retinopathy Study circle. Thickness of each layer was compared between groups, and the sectors with better area under the receiver operating characteristic curve (AUC) were identified.

**Results:**

mGCL was significantly thinner in the POAG group, especially in outer and inner temporal sectors (p<0.001); and mRNFL was significantly thinner in the POAG group in the outer inferior and the outer superior sector (p<0.001). Diagnostic accuracy of inner macular layers was good, and in general mGCL was superior to mRNFL. pRNFL obtained the best diagnostic capability (AUC, 0.886). Horizontal and vertical Posterior Pole protocols performed similarly.

**Conclusions:**

Inner macular layers using either horizontal or vertical B-scans, especially temporal sectors of mGCL, have good diagnostic capability to differentiate early glaucomatous eyes from control eyes; however, pRNFL has the highest diagnostic sensitivity for glaucoma detection.

## Introduction

The advent of new spectral domain optical coherence tomography (SD-OCT) technology has allowed a more rapid acquisition of retinal images at a higher axial-image resolution, enabling better identification of individual retinal layers [1]. Consequently, there has been an increasing interest in the importance of evaluating inner macular layers in glaucoma diagnosis [2]. Recent studies have demonstrated that macular damage occurs early in the disease process [3] and structural changes in the macula can thus precede detectable visual field (VF) loss [4].

It has been established that inner macular layers are more effective than total macular thickness in discriminating glaucoma patients [5,6]. Currently there are different OCT instruments available with different retinal segmentation algorithms. Some of them such as Cirrus HD-OCT (Carl Zeiss Meditec, Jena, Germany) delimit together ganglion cell layer and inner plexiform layer (GCL-IPL). Others like RTVue SD-OCT (Optovue, Inc., Fremont, CA) segment together macular retinal nerve fiber layer (mRNFL) and GCL-IPL, which is the called ganglion cell complex (GCC). The new software of the Spectralis OCT (Heidelberg Engineering, Inc., Heidelberg, Germany) is the first one able to measure each of the 10 histological retinal layers individually.

Previous studies have showed that the typical glaucomatous changes are located predominantly in the temporal macular regions along the horizontal raphe, usually in the same hemifield as the corresponding peripapillary retinal nerve fiber layer (pRNFL) defect; and especially in inferior macula [6–9]. Moreover, macular ganglion cell imaging shows less variability than conventional pRNFL and optic disc parameterS [10]; and it is less affected by age and optic disc size [11]. All these findings suggest the potential ability of inner macular layers to detect early glaucoma, using the layer thicknesses of the macular zones affected earlier in the glaucomatous neuropathy.

Based on the possibility of individual segmenting of the macular ganglion cell layer (mGCL) and the mRNFL, our purpose was to evaluate the diagnostic ability of these two layers in glaucomatous neuropathy, as well as to determine the macular areas in glaucoma eyes affected earlier. To our knowledge, this is the first study to compare the ability of new automatic segmentation software for Spectralis SD-OCT in the macular area, with both horizontal and vertical scan Posterior Pole protocols.

## Materials and methods

### Study population and design

This was an observational, prospective cross-sectional study. Participants were consecutively enrolled from the Glaucoma Department at the Miguel Servet University Hospital (Zaragoza, Spain). Age-and sex-matched healthy subjects who visited the ophthalmology outpatients department during the recruitment period, were included in the control group. Written informed consent was obtained from all participants after an explanation of the nature and potential consequences of the research. The study protocol observed the tenets of the Declaration of Helsinki, and was approved by the Clinical Regional Ethics Committee of Aragón (CEICA).

Inclusion criteria included a clinical diagnosis of primary open-angle glaucoma (POAG) at a previous visit at least 1 year before. Diagnosis of POAG was based on characteristic optic nerve damage on slit-lamp examination (defined as a definite notch in the neuroretinal rim or absence of the neuroretinal rim not due to another known cause) with corresponding VF defects, an open-appearing anterior chamber angle, and increased intraocular pressure (IOP, >21 mmHg). A glaucomatous VF defect was defined as the presence of 3 or more significant (p<0.05) non-edge continuous points with at least 1 at the p<0.01 level on the same side of the horizontal meridian in the pattern deviation plot, and classified as “outside normal limits” on the Glaucoma Hemifield Test, confirmed on two consecutive VF examinations. We selected for this study only patients with early glaucoma, according to the Hodapp-Parrish-Anderson criteria, with mean deviation between 0 and -6 Db [12].

All healthy subjects recruited had healthy-looking optic disc, IOP ≤21 mmHg in both eyes, VF within normal limits, no previous history of intraocular disease or surgery, and no family history of glaucoma.

Subjects from both groups (POAG and control group) were excluded if they had vision loss secondary to another eye condition, had undergone any laser procedure in the previous 2 months, or any ocular surgery in the previous 3 months. Other exclusion criteria included extreme refractive errors, such as high myopia (-6.0 or higher), hyperopia (+6.0 or higher), or astigmatism (±3.0 or higher), acute angle closure glaucoma, and evidence of macular pathologies, vascular or inflammatory diseases, or optic nerve neuropathies other than glaucoma. Patients with clinically significant lenticular opacity using the LOCS III classification [13] were also excluded. The exclusion criteria for lenticular opacity were nuclear color/opalescence greater than NC2 and NO2, respectively, cortical cataract greater than C2, and posterior subcapsular cataract greater than or equal to P1. One eye of each participant was included in this study according to the eligibility criteria described. If both eyes met the eligibility criteria, one eye was selected randomly.

### Ophthalmologic examination

We consecutively recruited 90 subjects: 45 healthy controls and 45 early glaucoma patients. The ocular examinations were conducted between January and June 2017, and included measurements of best-corrected visual acuity (using a Snellen chart at 4 m) and IOP (using a calibrated Goldmann applanation tonometer), slit lamp examination of the anterior segment, and fundus evaluation. The Humphrey 24–2 Swedish Interactive Threshold Algorithm Standard perimeter (Zeiss Meditec, Dublin, CA) was used to evaluate VF. Only reliable VFs were used, defined as those with <20% fixation errors and <33% false positives or false negatives. VF examinations were performed within 2 months of the OCT measurements.

### Spectral-Domain optical coherence tomography imaging

All participants were examined using pRNFL Glaucoma protocol, and horizontal and vertical Posterior Pole protocols of the Spectralis OCT. Images needed to have a quality index of at least 20 to be included in the study. Images with artifacts were excluded.

Images were obtained using the automated eye alignment eyetracking software (TruTrack; Heidelberg Engineering) to acquire perifoveal volumetric retinal scans comprising 61 horizontal single lines with 15 frames on average (30° × 25° volume scan centered at the fovea) and 19 vertical single lines with 15 frames on average (30° × 15° volume scan centered at the fovea) applying the horizontal and vertical Posterior Pole protocols, respectively. These two protocols include the Anatomic Positioning System (APS), which provides an anatomic map of each patient´s eye using two fixed structural landmarks: the center of the fovea and the center of Bruch´s membrane opening. With APS, all scan protocols are automatically established according to the patient anatomic map. This enables accurate examination of relevant structures and ensures precise comparisons with reference data.

The pRNFL was scanned using the circular scan mode of the Spectralis OCT system, which consisted of 768 A-scans. The scan circle subtended 12°, and the diameter in millimeters depended on the axial lengths. Correction for fovea-disc orientation was provided automatically by the software with the Fovea-Disc Alignment system. In all cases, foveal fixation and segmentation were controlled to be correct.

**Fig 1:**
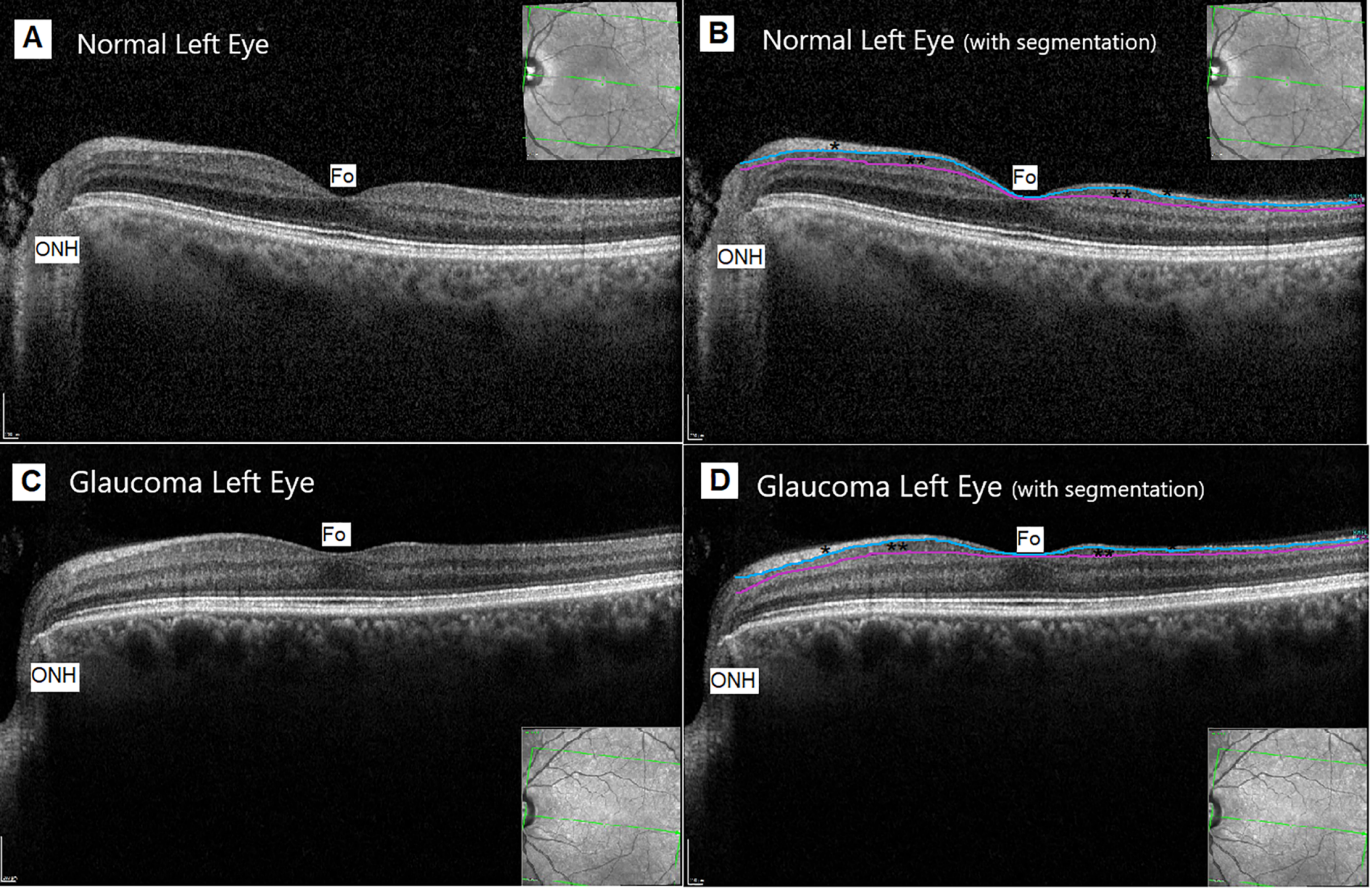
**Representative optical coherence tomography (OCT) horizontal scan section of the macula of a normal (A and B) and early glaucomatous (C and D) left eye**. Macular ganglion cell layer (mGCL) is marked with two asterisks, and retinal nerve fiber (mRNFL) layer is marked with one asterisk, in Fig 1B (normal eye) and 1D (glaucomatous eye). The automated segmentation performed by the OCT Spectralis software between mRNFL and mGCL is shown with a light blue line, and between mGCL and inner plexiform layer is shown with a purple line. We can appreciate a slight thinning of mGCL in the glaucomatous eye, especially temporal to fovea. Optic nerve head (ONH) position and fovea (Fo) are indicated. The infrared image obtained with the Horizontal Posterior Pole protocol of Spectralis OCT is shown in the corner of each B-scan. The green lines of the infrared image delimit the square scanning area at the posterior pole.

**Fig 2:**
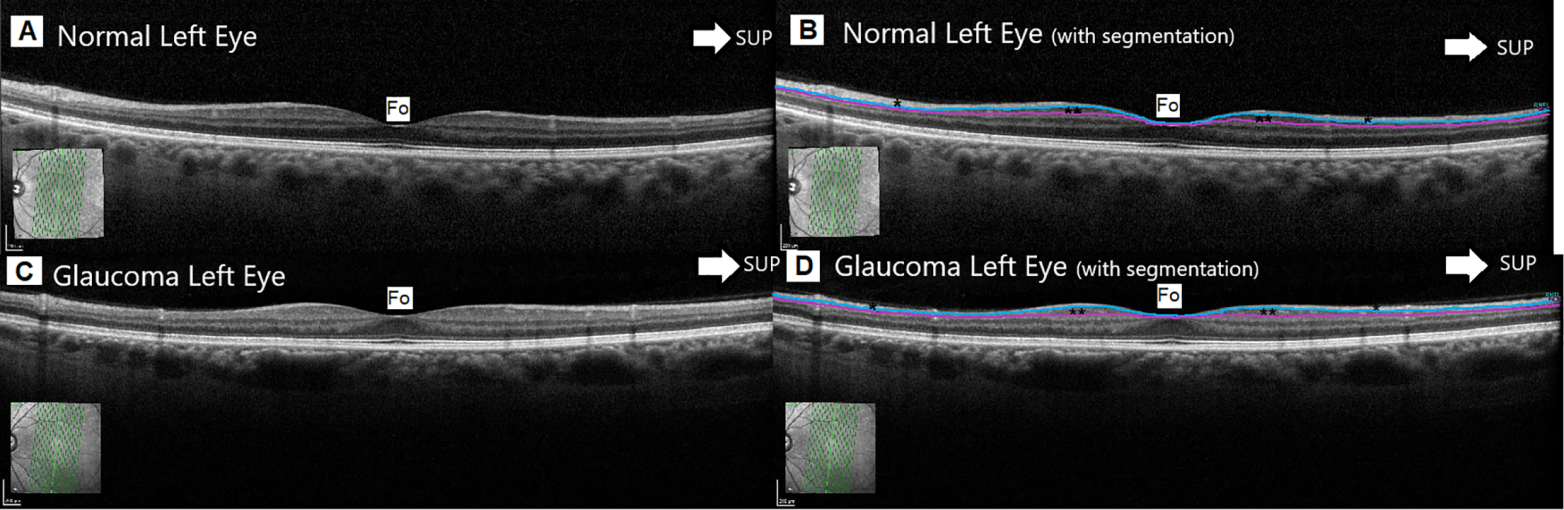
**Representative optical coherence tomography (OCT) vertical scan section of the macula of a normal (A and B) and early glaucomatous (C and D) left eye**. Macular ganglion cell layer (mGCL) is marked with two asterisks, and retinal nerve fiber (mRNFL) layer is marked with one asterisk, in Fig 1B (healthy eye) and 1D (glaucomatous eye). The automated segmentation performed by the OCT Spectralis software between mRNFL and mGCL is shown with a light blue line; and the one between mGCL and inner plexiform layer is shown with a purple line. The arrow indicates superior zone, and fovea position (Fo) is also pointed out. We can appreciate a slight thinning of mRNFL in the glaucomatous eye, especially inferiorly to the fovea. The infrared image obtained with the Vertical Posterior Pole protocol of Spectralis OCT is shown in the lower left corner of each B-scan. The green lines of the infrared image delimit the square scanning area at the posterior pole.

Adopting these specific protocols, images were obtained by an experienced operator (B.C.) and all the images were reviewed thoroughly by a glaucoma specialist (M.P.B.) who was concealed to clinical information and then assessed quality, alignment, and artifacts. Layer-by-layer segmentation was carried out automatically in this instrument using the new software for the Spectralis OCT Heidelberg Eye Explorer 6.8a (Figs 1 and 2), and it was checked to be adequate in the 61 horizontal B-scans and in the 19 vertical B-scans of each imaged eye applying the criteria of Ishikawa et al.[14] as a reference. In detail, we excluded 3 eyes with incorrigible segmentation failures; 2 eyes with low-quality signal strength; and 2 eyes due to artifacts.

**Fig 3:**
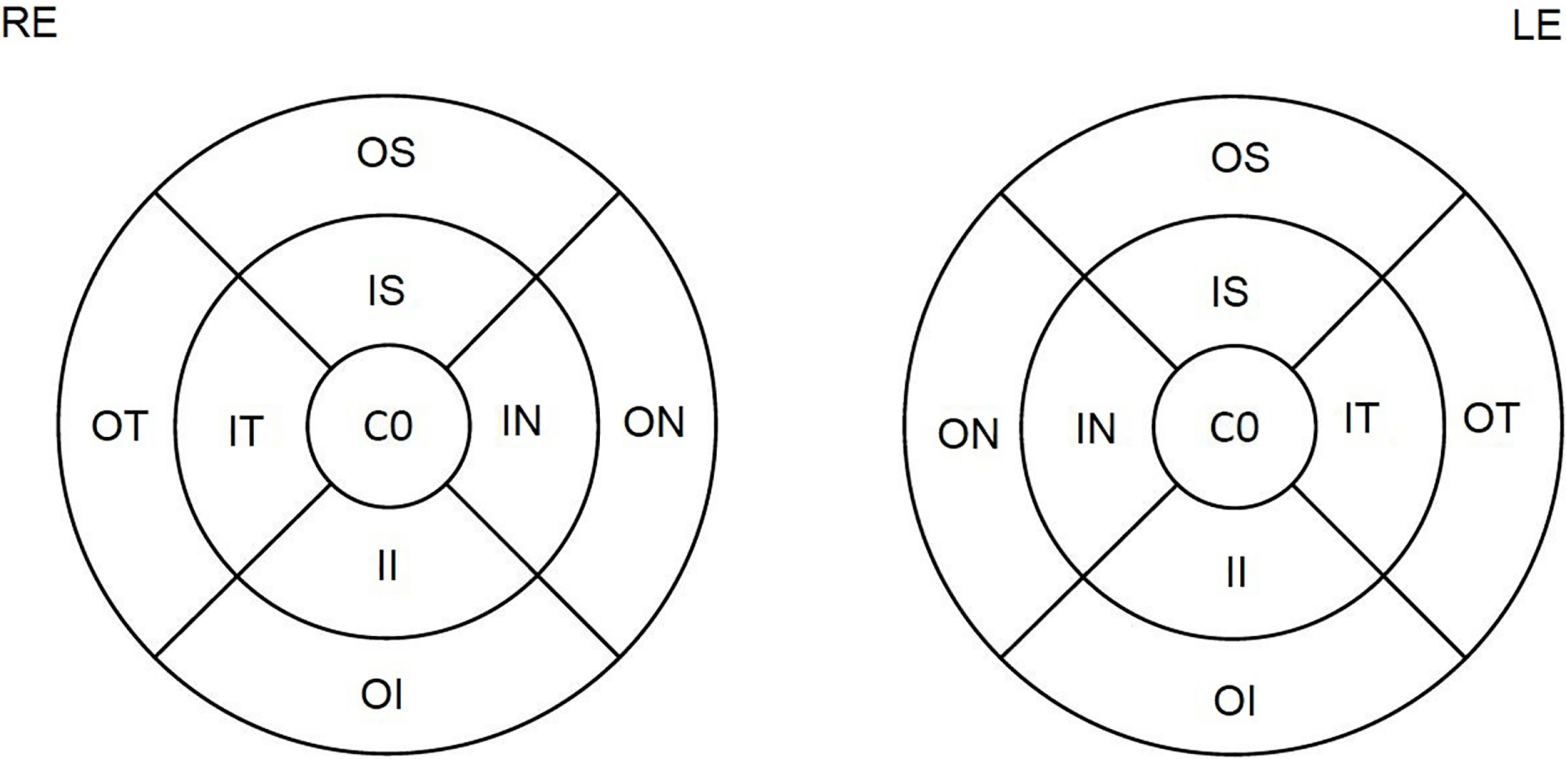
Early treatment diabetic retinopathy study (ETDRS) subfields. Macular ganglion cell (mGCL) and retinal nerve fiber (mRNFL) layers were measured in each of the nine macular areas defined by the ETDRS circle. Abbreviation: C0, central fovea; IS, inner superior; IN, inner nasal; II, inner inferior; IT, inner temporal; OS, outer superior; ON, outer nasal; OI, outer inferior; and OT, outer temporal; RE, right eye; LE, left eye.

We used retinal thickness map analysis to represent numeric averages of the measurements for each of the nine Early Treatment Diabetic Retinopathy Study (ETDRS) subfields. The inner, intermediate, and outer rings centered at the fovea, with diameters of 1, 3, and 6 mm, respectively, were acknowledged for the analysis. The average thicknesses of the following nine zones were used in the analysis: central fovea, inner superior, inner nasal, inner inferior, inner temporal, outer superior, outer nasal, outer inferior and outer temporal (Fig 3). Macular RNFL and mGCL were measured in each of these 9 macular areas determined by the ETDRS circle described above, and used in the analysis. Global thickness and mean pRNFL in each of the 6 studied zones were also recorded: superotemporal, superonasal, nasal, inferonasal, temporal, and inferotemporal.

### Statistical analysis

Baseline descriptive characteristics were compared between healthy and POAG groups using an unpaired Student t test for quantitative variables (after checking normality in the distribution) and the Chi-square test for categorical variables (sex). Then, mean thickness in different layers was compared between the groups in each sector using an unpaired Student t test. The layers compared were the standard pRNFL, mRNFL, and mGCL.

The diagnostic accuracy of each parameter in each layer (in terms of statistical significance and effect measure) to differentiate between healthy and diseased eyes was calculated by means of the area under the receiver operating characteristics (ROC) curve (AUC). The curves show the values at different levels of sensitivity (ability to detect truly diseased patients) on the y-axis and 1-specificity (false-positive rate) on the x-axis for each parameter. The AUC summarizes the global value of the parameter, in which values closer to 1 represent higher diagnostic discriminant ability. The following established five-category rating scale was used for interpreting AUC values: >0.90, excellent, 0.80–0.90, good; 0.70–0.80, fair; 0.60–0.70, poor; and 0.50–0.60, fail [15].

Finally, the relationship between functional (as measured through mean deviation -MD- loss on the VF in decibels) and structural loss (mean thickness for pRNFL and volume for mRNFL and mGCL) in all participants was determined using Pearson´s correlation coefficient.

Sample size calculations were performed and the obtained results were that 40 participants were needed in each group to find statistically significant differences, so we initially recruited 45 controls, and 45 POAG.

The method of Bonferroni was used for multiple comparisons of mean layer thickness between healthy and POAG eyes. All data analyses were performed using SPSS version 20.0 (SPSS Incorporation, Chicago, IL) statistical software.

## Results

Analysis was finally performed on 40 early POAG eyes of 40 participants and 43 healthy eyes of 43 normal participants. The demographic and clinical characteristics and pRNFL thicknesses of each group are summarized in Table 1. There were no differences in age, gender and spherical equivalent between groups. As expected, MD was significantly worse in the glaucoma group (MD, -3.06±1.86 dB) than in normal participants (MD, -0.33±1.47 dB).

**Table 1:** Descriptive and clinical data and mean and standard deviation of peripapillary retinal nerve fiber layer thickness, for 40 early open-angle glaucoma eyes and 43 healthy control eyes, and comparison between groups.

	CONTROL	POAG	P^a^
Number of eyes	43	40	-
Age [years]	64.67±5.24	67.43±8.93	0.099
Gender [male/female]	13/30	19/21	0.106
Spherical Equivalent [D]	0.51±1.48	0.04±2.43	0.309
BCVA [Snellen]	0.97±0.06	0.89±0.17	0.007
IOP [mm Hg]	16.41±2.25	16.85±3.59	0.513
MD [dB]	-0.33±1.47	-3.06±1.86	**<0.001**
Corneal pachymetry [μm]	563.62±30.15	538.55±38.59	**0.002**
pRNFL Global [μm]	94.44 ± 9.68	75.92 ± 12.55	**<0.001**
pRNFL Temporal [μm]	65.51 ±8.06	57.52 ± 12.53	**0.001**
pRNFL Temporal-Superior [μm]	133.46 ± 17.34	96.22 ± 24.29	**<0.001**
pRNFL Temporal-Inferior [μm]	138.11 ± 18.39	100.15 ± 28.74	**<0.001**
pRNFL Nasal [μm]	70.39 ± 13.42	61.40 ± 16.52	0.008
pRNFL Nasal-Superior [μm]	103.04 ± 20.50	83.37 ± 20.44	**<0.001**
pRNFL Nasal-Inferior [μm]	109.02 ±21.42	89.70 ±24.03	**<0.001**

POAG, primary open-angle glaucoma; D, diopters; BCVA, best corrected visual acuity; IOP, intraocular pressure; MD, mean deviation; dB, decibels; pRNFL, peripapillary retinal nerve fiber layer.

^a^ p: level of statistical significance in comparison between the two groups using Student’s t-test (and chi-square test for gender).

Data are mean ± standard deviation. **Bold text** indicates statistically significant results using the Bonferroni correction for multiple comparison (**p<0.004**).

The comparison of the mean thickness of mGCL and mRNFL in each sector of the ETDRS circle (and total volume, center pixel, minimal and maximal thickness in the central circle sector of the measuring grid) between groups is shown in Table 2 (for mGCL) and Table 3 (for mRNFL).

**Table 2:** Mean and standard deviation of macular ganglion cell layer thickness obtained with horizontal and vertical scans in the control group and early glaucoma patients, and comparison between groups.

		CONTROL	POAG	P^a^
Total volume (mm^2^)	Post Pole H	1.02 ± 0.08	0.89 ± 0.14	**<0.001**
	Post Pole V	1.03 ± 0.12	0.93 ± 0.15	**<0.001**
Central circle [μm]	Post Pole H	14.67 ± 3.72	13.20 ± 3.93	0.083
	Post Pole V	14.53 ± 3.73	13.15 ± 4.05	0.111
Inner nasal [μm]	Post Pole H	48.81 ± 5.26	45.98 ± 9.08	**0.001**
	Post Pole V	49.55 ± 5.22	44.10 ± 9.30	**0.001**
Outer nasal [μm]	Post Pole H	36.67 ± 3.58	34.55 ± 5.50	0.039
	Post Pole V	39.97 ± 4.03	37.33 ± 6.11	0.022
Inner superior [μm]	Post Pole H	50.05 ± 4.46	43.48 ± 9.20	**<0.001**
	Post Pole V	49.55 ± 4.38	44.05 ± 9.06	**0.001**
Outer superior [μm]	Post Pole H	32.39 ± 2.54	29.95 ± 4.57	0.003
	Post Pole V	32.67 ± 2.50	30.02 ± 4.70	0.002
Inner temporal [μm]	Post Pole H	45.65 ± 4.71	36.58 ± 8.97	**<0.001**
	Post Pole V	45.95 ± 5.20	37.53 ± 9.14	**<0.001**
Outer temporal [μm]	Post Pole H	32.67 ± 3.01	26.85 ± 5.05	**<0.001**
	Post Pole V	35.72 ± 3.93	29.56 ± 5.74	**<0.001**
Inner inferior [μm]	Post Pole H	49.88 ± 4.59	43.57 ± 8.37	**<0.001**
	Post Pole V	49.69 ± 4.45	43.46 ± 8.65	**<0.001**
Outer inferior [μm]	Post Pole H	31.00 ± 2.13	27.25 ± 4.67	**<0.001**
	Post Pole V	31.30 ± 2.35	27.71 ± 4.95	**<0.001**
Center [μm]	Post Pole H	2.25 ± 2.02	2.67 ± 1.77	0.320
	Post Pole V	2.25 ± 3.23	1.71 ± 2.30	0.393
Central Min [μm]	Post Pole H	1.44 ± 1.97	1.67 ± 1.26	0.528
	Post Pole V	1.13 ± 2.06	0.87 ± 1.43	0.502
Central Max [μm]	Post Pole H	34.16 ± 6.75	31.40 ± 9.81	0.137
	Post Pole V	35.25 ±6.49	31.58 ± 8.88	0.035

POAG, primary open-angle glaucoma; Center, center pixel of the measuring grid; Central Min, minimal retinal thickness in the central circle sector; Central Max, maximal retinal thickness in the central circle sector; Post Pole H, posterior pole protocol with horizontal scans; Post Pole V, posterior pole protocol with vertical scans.

^a^p: level of statistical significance in comparison between the two groups using Student’s t-test. Data are mean ± standard deviation. **Bold text** indicates statistically significant results using the Bonferroni correction for multiple comparison (**p<0.002**).

**Table 3:** Mean and standard deviation of macular retinal nerve fiber layer thickness obtained with horizontal and vertical scans in the control group and early glaucoma patients, and comparison between groups.

		CONTROL	POAG	P^a^
Total volume (mm^2^)	Post Pole H	0.86 ± 0.15	0.77 ± 0.13	0.008
	Post Pole V	0.83 ± 0.09	0.73 ± 0.12	**<0.001**
Central circle [μm]	Post Pole H	12.20 ± 1.71	11.50 ± 2.54	0.137
	Post Pole V	12.23 ± 2.05	11.27 ± 2.83	0.083
Inner nasal [μm]	Post Pole H	20.53 ± 2.83	19.75 ± 2.15	0.162
	Post Pole V	19.73 ± 2.65	18.70 ± 2.03	0.025
Outer nasal [μm]	Post Pole H	46.16 ± 6.50	40.87 ± 9.45	0.004
	Post Pole V	41.25 ± 4.99	35.45 ± 7.70	**<0.001**
Inner superior [μm]	Post Pole H	23.30 ± 3.33	21.35 ± 2.68	0.004
	Post Pole V	23.30 ± 3.40	20.97 ± 2.72	**0.001**
Outer superior [μm]	Post Pole H	35.93 ± 4.26	30.17 ± 5.93	**<0.001**
	Post Pole V	35.18 ± 4.14	30.05 ± 6.71	**<0.001**
Inner temporal [μm]	Post Pole H	17.51 ± 2.25	18.77 ± 2.51	0.018
	Post Pole V	17.02 ± 2.36	17.32 ± 1.54	0.510
Outer temporal [μm]	Post Pole H	19.13 ± 1.03	18.80 ± 2.28	0.381
	Post Pole V	19.09 ± 1.01	18.16 ± 1.74	0.004
Inner inferior [μm]	Post Pole H	24.46 ± 3.46	23.15 ± 3.96	0.111
	Post Pole V	24.37 ± 3.16	22.43 ± 3.72	0.014
Outer inferior [μm]	Post Pole H	38.16 ± 4.07	30.30 ± 9.89	**<0.001**
	Post Pole V	37.30 ± 4.61	30.21 ± 9.57	**<0.001**
Center [μm]	Post Pole H	1.30 ± 2.25	1.41 ± 2.25	0.829
	Post Pole V	0.93 ± 2.68	1.13 ± 2.59	0.731
Central Min [μm]	Post Pole H	0.93 ± 2.09	0.58 ± 1.18	0.375
	Post Pole V	0.60 ± 2.17	0.35 ± 1.37	0.543
Central Max [μm]	Post Pole H	24.02 ± 3.89	23.15 ± 4.45	0.349
	Post Pole V	25.27 ± 3.81	24.72 ± 4.06	0.535

POAG, primary open-angle glaucoma; Center, center pixel of the measuring grid; Central Min, minimal thickness in the central circle sector; Central Max, maximal thickness in the central circle sector; Post Pole H, posterior pole protocol with horizontal scans; Post Pole V, posterior pole protocol with vertical scans.

^a^ p: level of statistical significance in comparison between the two groups using Student’s t-test). Data are mean ± standard deviation. **Bold text** indicates statistically significant results using the Bonferroni correction for multiple comparison (**p<0.002**).

Peripapillary RNFL was significantly thinner in the POAG group globally and in all sectors assessed except nasal sector (p<0.001). For the macular parameters, mGCL thickness was reduced significantly in the POAG group for the following sectors (p<0.001): total volume, inner nasal, inner superior, inner temporal, outer temporal, inner inferior and outer inferior; with both horizontal and vertical scan Posterior Pole protocols. Inner temporal sector showed the maximum difference between groups (9.07μm of difference with horizontal scan protocol, and 8.52μm of difference with vertical scan protocol).

Macular RNFL thickness was reduced significantly in the POAG group for the following sectors (p<0.001): total volume, outer nasal and inner superior (with vertical scan protocol); outer superior and outer inferior (with vertical and horizontal scan protocols). Outer inferior sector showed the maximum difference between groups (7.86μm of difference with horizontal scan protocol, and 7.09μm with vertical scan protocol).

**Fig 4:**
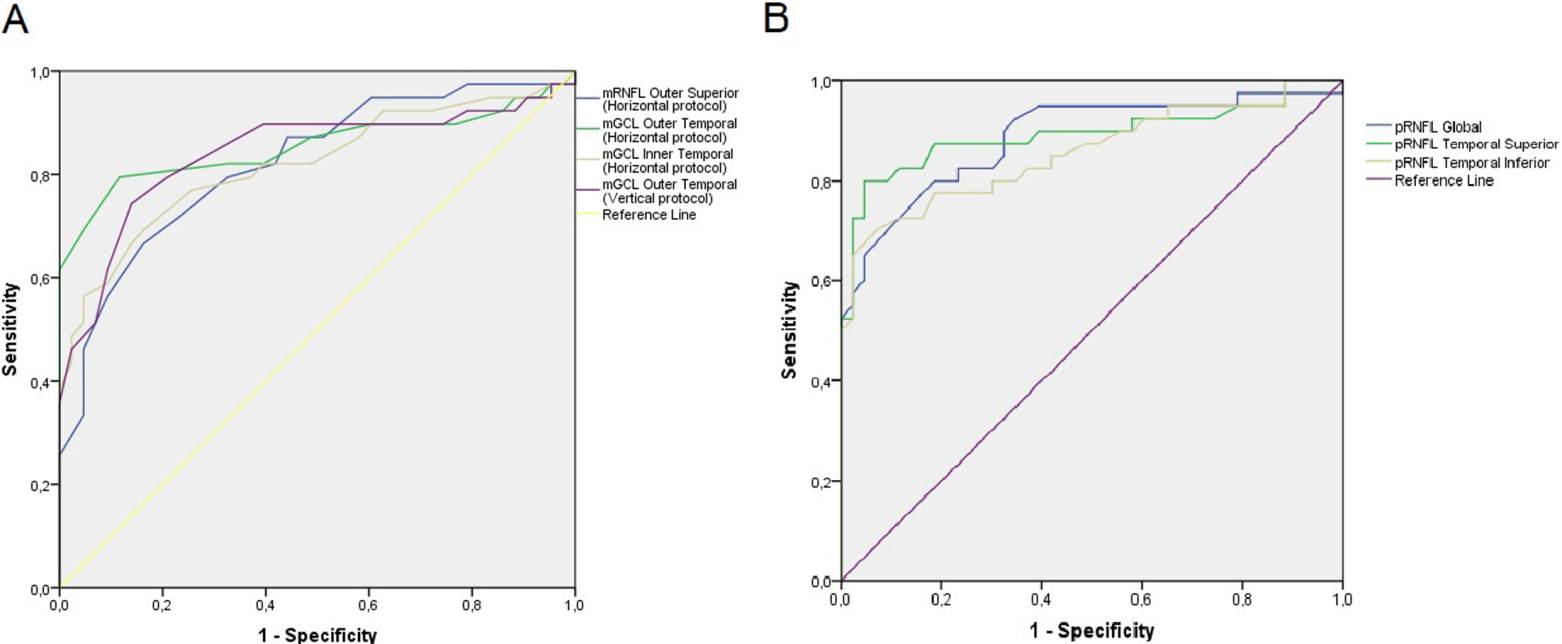
**Receiver operating characteristic (ROC) curves for the macular (A) and peripapillary (B) parameters with the greater discriminating ability.** Abbreviations: mRNFL, macular retinal nerve fiber layer; mGCL, macular ganglion cell layer; pRNFL, peripapillary retinal nerve fiber layer.

Table 4 shows the values for AUC of the best parameters analyzed. The best parameters were global thickness of pRNFL (AUC, 0.886), the temporal-superior sector of pRNFL (AUC, 0.893), the temporal-inferior sector of pRNFL (AUC, 0.856), the outer superior sector of mRNFL with horizontal scan Posterior Pole protocol (AUC, 0.806), the inner and outer temporal sectors of mGCL with horizontal scan Posterior Pole protocol (AUC, 0.823 and 0.858, respectively), and the outer temporal sector of mGCL with vertical scan Posterior Pole protocol (AUC, 0.840). Fig 4A shows ROC curves and corresponding AUCs for the best macular parameters; and Fig 4B shows ROC curves and corresponding AUCs for the best peripapillary RNFL parameters.

**Table 4:** Area under the receiver operating characteristics curve (AUC) of the most relevant parameters on each layer.

PARAMETER	AUC (95% Confidence Interval)
pRNFL/ Global	**0.886** (0.811–0.961)
pRNFL/ Temporal- Superior	**0.893** (0.815–0.971)
pRNFL/ Temporal-Inferior	**0.856** (0.771–0.941)
pRNFL/ Temporal- Superior	0.772 (0.667–0.877)
pRNFL/ Nasal-Inferior	0.727 (0.617–0.837)
mRNFL (H) / Total Volume	0.749 (0.637–0.860)
mRNFL (H) / Outer Superior	**0.806** (0.711–0.901)
mRNFL (H) / Outer Inferior	0.783 (0.672–0.894)
mRNFL (V) Total Volume	0.781 (0.673–0.888)
mRNFL (V) Outer Superior	0.764 (0.656–0.873)
mRNFL (V) Outer Inferior	0.795 (0.686–0.905)
mGCL (H) Total Volume	0.799 (0.696–0.902)
mGCL (H) Inner Temporal	**0.823** (0.729–0.917)
mGCL (H) Outer Temporal	**0.858** (0.767–0.950)
mGCL (V) Total Volume	0.759 (0.649–0.870)
mGCL (V) Inner Temporal	0.791 (0.690–0.892)
mGCL (V) Outer Temporal	**0.840** (0.746–0.935)

pRNFL, peripapillary retinal nerve fiber layer; mRNFL, macular retinal nerve fiber layer; H, horizontal posterior pole protocol; V, vertical posterior pole protocol; mGCL, macular ganglion cell layer. **Bold text** indicates AUC > 0.8 (good diagnostic ability).

Correlations between functional loss (MD) and structural loss (mean thickness for pRNFL and volume for mRNFL and mGCL) were slightly statistically significant (r coefficient ranged between 0.48 and 0.51, p<0.001).

## Discussion

The present study analyzed the diagnostic ability of two individual inner macular layers (mRNFL and mGCL) obtained using a new segmentation software, with Posterior Pole protocols (one performed with 61 horizontal B-scans and the other with 19 vertical B-scans) of Spectralis SD-OCT. Previous authors had evaluated the role of macular inner layers in glaucoma, but using horizontal scanning protocols only [4–7] or after a manual segmentation of SD-OCT B-scans [16]. This is the first study to compare the ability of new automatic segmentation software for Spectralis SD-OCT in the macular area, with both horizontal and vertical scan Posterior Pole protocols. The results showed that some sectors of inner macular layers have similar diagnostic capability than the classical peripapillary RFNL to differentiate early glaucomatous eyes from control eyes, and outer ETDRS sectors performed better than inner sectors for diagnosis. In general, mGCL was superior to mRFNL to discriminate glaucoma subjects, but pRNFL shown the best diagnostic accuracy.

We have found that mGCL is thinned in early glaucomatous eyes, especially in temporal and inferior sectors; and mRFNL also showed differences in outer superior and outer inferior sectors between early glaucomatous and control eyes. Results obtained for both layers with vertical and horizontal B-scans were very similar. These results are coincident to other author’s findings. For example, Nakano et al. [16] found a thinning of mGCL temporally and inferiorly in eyes with preperimetric glaucoma, and mGCL exhibited higher sensitivity than the other macular layers, particularly on vertical scans after a manually segmentation of SD-OCT B-scans.

In this study, we found that global thickness of pRNFL (AUC, 0.886) performed similar than outer temporal sector of mGCL (AUC, 0.858), although pRNFL parameters still continue being superior in general for diagnosis, with greater AUCs than inner macular layers. In this sense, two recent systematic reviews have concluded that pRNFL parameters are still preferable to macular parameters for diagnosing manifest glaucoma, but the differences were small [17,18]. However, more recently Kim et al.[19] using the same new segmentation software of Spectralis OCT, found that mRNFL (AUC, 0.915) and mGCL (AUC, 0.914) had the best AUCs and not statistically different from pRNFL (AUC, 0.878), but they included GPAA with different disease severity.

The macular parameters with better AUCs in our study were temporal sectors for mGCL, and outer superior and inferior sectors of mRNFL. This is consistent with previous studies such us Lee et al.[20] who showed that AUC was better for the GC-IPL in outer temporal sectors; or Nakano et al.[16] or Nakatani et al. [6] who demonstrated outer macular zones in general have better diagnostic ability than inner zones. This is due to the fact that most of the retinal ganglion cells in the inferior macular region project their axons to the inferior optic nerve head (ONH) quadrant. Therefore, it is expected that glaucomatous damage that affects especially to the inferior pole of the ONH, results in damage to the inferior and inferotemporal macular zones [8,21].

On the other hand, we found the thinning of mGCL of early glaucomatous eyes were located in different areas than mRNFL: only the outer superior and the outer inferior sectors of mRNFL were statistically different with horizontal and vertical scan Posterior Pole protocols, and not in the temporal region. This could be explained by mRNFL being in general thinner in the outer temporal zone than in other macular zones, thus making differences difficult to be detected with segmentation algorithms. And this may also be attributable to the spatial discrepancy between the axons and their original retinal ganglion cell bodies [8,22]. We found in general better AUCs with mGCL than mRNFL. However other authors, like Pazos et al. [23] evaluated diagnostic accuracy of this new segmentation software in early glaucomatous eyes, and they concluded mRNFL isolated showed a high ability (AUC outer inferior sector, 0.906), while mGCL showed less diagnostic ability (AUC inner temporal sector, 0.858); but in general, pRNFL still performed better than macular layers (AUC, 0.956). In another previous study with a preliminary version of this segmentation software, Martinez de la Casa et al. [24] found that mRFNL (AUC, 0.742) was the best discriminating parameter even compared with pRNFL (0.595), but in that case compared with glaucoma suspects, not established glaucoma.

Discrepancies observed in different studies regarding the macular inner layers most affected by glaucoma (mGCL, GCL-IPL or mRNFL), may be due to several reasons. First, the possible variability in the selected glaucoma sample: glaucoma suspects in some studies [24] and mild glaucoma in others [23]. Secondly, differences could be due to the possible variability during the OCT automated segmentation process. This variability in OCT segmentation could be increased if we analyze studies using different devices, since the OCT axial resolution is different, as well as the algorithms used for segmentation.

Finally, in our early glaucomatous eyes (mean MD, -3.06 dB), the association of VF MD with global structural measurements (mean thickness for pRNFL, and volume for mRNFL and mGCL), were statistically significant but slight (r ~ 0.5). These results are similar to those reported in other studies [23,25].

This study has several limitations. First, the lack of a normative database for the Spectralis new segmentation software does not permit us neither to calculate how many patients were in or out of the normal range, nor to compare results with those obtained in pRNFL analysis of the same patients. Secondly, subjects with a subclinical macular disease could have been included; however, we applied strict inclusion and exclusion criteria, so longitudinal follow-up was necessary to ratify our results. Thirdly, a reproducibility study to assess repeatability of the segmentation software was not performed. Fourth, we only include early glaucomatous eyes, and it could be interesting to evaluate the same macular parameters in moderate and severe POAG, to determine the principal zones and layers affected when disease progresses. And fifth, the relative small sample size and homogeneous ethnicity (all were Caucasians), make necessary to develop new studies with bigger and heterogeneous population.

Macular ganglion cell analysis may avoid some limitations of peripapillary region, such as interference from retinal and optic nerve head vasculature, circumpapillary atrophy, and variable placement of the measurement circle around the optic disc. But clinicians should not rely entirely on macular parameters, because in addition to glaucoma, other macular diseases (such us diabetic retinopathy, macular edema, macular degeneration, and epiretinal membranes) are also common in aging population, and this condition may affect OCT macular thickness measurements. Thus, we consider that a combination of peripapillary and macular parameters is probably the best approach for glaucoma detection.

In conclusion, we believe that inner macular layers, especially temporal sectors of mGCL, evaluated with the new segmentation software of Spectralis OCT have good ability to diagnose incipient glaucoma, using either horizontal or vertical B-scan Posterior Pole protocol.
